# Physiological and Anthropometric Determinants of Performance Levels in Professional Futsal

**DOI:** 10.3389/fpsyg.2020.621763

**Published:** 2021-01-12

**Authors:** Damir Sekulic, Haris Pojskic, Ivan Zeljko, Miran Pehar, Toni Modric, Sime Versic, Dario Novak

**Affiliations:** ^1^Faculty of Kinesiology, University of Split, Split, Croatia; ^2^Department of Sports Sciences, Linnaeus University, Kalmar, Sweden; ^3^Faculty of Science and Education, University of Mostar, Mostar, Bosnia and Herzegovina; ^4^Faculty of Kinesiology, University of Zagreb, Zagreb, Croatia; ^5^Institute for Anthropological Research, Zagreb, Croatia

**Keywords:** conditioning capacities, kicking speed, reactive agility, change of direction speed, team sports, fitness level, reactive strength index

## Abstract

There is an evident lack of studies examining the pursuit of excellence in futsal. The aims of this study were to evaluate anthropometric and physiological variables that may contribute to distinguishing among performance levels in professional futsal players and to evaluate correlates of those variables. The participants were 75 male professionals (age = 25.1 ± 5.1 years, body height = 182.3 ± 6.2 cm, body mass = 80.8 ± 10.4 kg), who were divided into performance levels using two criteria: (i) starters (first teams) vs. non-starters (substitutes) and (ii) top-level players (members of the national team and players who participated in top-level team competition in Europe) vs. high-level players (team players competing at the highest national competitive rank). Variables included anthropometrics (body height and mass, BMI, body fat percentage), generic tests of physiological capacities [5- and 10-m sprints, countermovement jump, broad jump, 20-yard test, reactive strength index (RSI)], and futsal-specific fitness tests [kicking speed by dominant and non-dominant leg, futsal-specific tests of change of direction speed, and reactive agility (FSRAG) involving/not involving dribbling the ball]. Top-level players outperformed high-level players in RSI, broad jump, kicking speed, and FSRAG involving dribbling. Starters achieved better results than non-starters in fewer variables, including kicking speed and RSI. Body fat percentage negatively influenced FSRAG involving dribbling, and RSI. FSRAG, RSI, and kicking speed were significantly correlated, indicating the similar physiological background of these capacities. The findings suggest that enhanced reactive strength and the ability to rapidly change direction speed in response to external stimulus while executing futsal-specific motor tasks (e.g., dribbling), along with players’ ability to kick the ball speedily, can be considered essential qualities required for advanced performance in futsal. Consequently, futsal strength and conditioning training should be targeted toward lowering relative body fat, maximizing lower-body reactive strength and including futsal-specific skills (e.g., dribbling, shooting) in reactive agility drills.

## Introduction

Futsal is a high-intensity intermittent sport that requires players to repeatedly engage in sequences of intense activities (e.g., sprinting, changes of direction, acceleration, deceleration kicking) on the futsal court (Spyrou et al., 2020). During the match, futsal players cover 3000–4500 m (Barbero-Alvarez et al., 2008), and 26% of the total distance covered is performed at a high-intensity level (Doğramacı and Watsford, 2006), with approximately 26 sprints per match (Caetano et al., 2015). Additionally, recent studies reported cardiovascular stress higher than 85% of maximum heart rate (HRmax) during more than 80% of actual playing time (Ayarra et al., 2018), with players reaching HRmax in most of the matches (Trabelsi et al., 2014). Moreover, several studies reported the blood lactate concentration to be over the lactate threshold (>4.0 mmol L^–1^) during futsal matches (Milioni et al., 2016; Dos-Santos, 2020). Accordingly, futsal requires a high level of energy from both the anaerobic and aerobic systems (Castagna and Alvarez, 2010). Furthermore, well developed speed, agility, muscle strength and power are important in execution of specific futsal performances (e.g., shooting, dribbling, passing, ball recovery) and movements (e.g., accelerations, decelerations, sprints, change of directions, jumps) (Young et al., 2002; Alvarez et al., 2009; Castagna et al., 2009; Junior et al., 2017; Ribeiro et al., 2020) and consequently, they are considered to be key indicators of overall performance in futsal matches (Galy et al., 2015; Junior et al., 2017).

Studies already attempted to describe the physiological characteristics of futsal players across the competitive levels and age groups (Junior et al., 2017). For example, Alvarez et al. (2009) investigated aerobic fitness in futsal players of different competitive levels and noted that VO2max may be considered a competitive-level dependent physical variable in futsal. Pedro et al. (2013) reported that running speeds at the ventilatory threshold and maximal oxygen consumption discriminate the competitive futsal level, with better performance in higher performance-levels. Nakamura et al. (2016) investigated differences in physical performance between U-20 and senior top-level Brazilian futsal players and reported that long-term exposure to futsal may lead to improvement in aerobic fitness and cardiac autonomic regulation while impairing the muscle power and speed performance. Recently, Sekulic et al. (2019) highlighted the importance of agility in identifying the performance levels of professional futsal players using newly developed tests of the change of direction speed (CODS) and reactive agility (RAG) in competitive futsal players. Finally, literature indicates that performance levels of futsal players differ in match running performances. In brief, elite futsal players cover greater total distance with higher intensities and perform a greater number of sprints during match-play when compared to sub-elite players (Spyrou et al., 2020).

It is widely accepted that anthropometric characteristics might be an important correlate of physical performance and factor of success in sport (Rienzi et al., 2000). For example, several studies suggested that high body mass and body fat measurements were related to poor muscle power in soccer (Nikolaïdis, 2012), basketball (Nikolaidis et al., 2015), and handball (Nikolaidis and Ingebrigtsen, 2013) players. On the other hand, anthropometric characteristics of futsal players have not been frequently investigated. In particular, Nikolaidis et al. (2019) examined the relationship between age and body mass status with field and laboratory measures of physical fitness in Greek futsal players at different age levels and concluded that the prevalence of overweight in futsal players should be an important concern for practitioners working in this team sport. Galy et al. (2015) assessed the anthropometric and physiological characteristics of Melanesian futsal players and found significant correlation between body height and countermovement jump (CMJ) performance. Recently, López-Fernández et al. (2020) investigated bilateral asymmetries between elite (1st league) and sub-elite (3rd league) male futsal players, and noted significant bilateral asymmetry in fat-mass percentage between dominant and non-dominant limbs for sub-elite players.

Although physiological and anthropometric characteristics of futsal players were extensively investigated, only few studies have focused on different competitive levels. These comparative analyses are important because they reveal physiological and anthropometric factors that differentiate players at different competitive and expertise levels (Naser and Ali, 2016). Specifically, Jiménez-Reyes et al. (2019) indicated that elite futsal players present better sprinting abilities when compared to lower-level players, but that jumping capacity seems not to differentiate between competition levels. Further, Ayarra et al. (2018) reported no significant differences in either acceleration capacity (5 and 15 m) or CODS ability between third and second division and junior players. However, those researchers found players competing at a higher playing level to have better jumping and sprinting abilities (Ayarra et al., 2018). Similarly, Naser and Ali (2016) reported that first division players were faster than amateur players over a 5 m distance.

The rationale of this study comes from the lack of knowledge of determinants of performance-level differences in professional futsal. More specifically, although, previous studies provided important insights into the physiological and anthropometric characteristics required to play at different competitive levels, it is evident that most of the previous investigations evidenced differences between relatively diverse performance levels (Naser and Ali, 2016; Ayarra et al., 2018; López-Fernández et al., 2020). In other words, only few studies determined the differences between performance groups by examining the pursuit of excellence, while to the best of our knowledge, no study has examined this issue in international samples. Supportively, in a very recent review paper examining the characteristics of the futsal demands and players’ characteristics authors noted that little is known regarding elite and sub-elite futsal players’ neuromuscular abilities (i.e., strength, jumping, sprinting, and change of direction) (Spyrou et al., 2020). This issue is particularly important for creating the profiles of players that can respond to the physical demands of the highest competition levels (i.e., champions’ league or national team competitions).

The main objectives of this study were to identify the fitness status and performance-level differences in an international sample of professional futsal players. Additionally, we studied the correlates of the most important fitness parameters in the studied players. An understanding of these characteristics is expected to be beneficial for coaches in the selection of players that are better suited to the highest competition levels. Originally, we hypothesized that fitness variables will be significant determinants of performance levels in professional futsal.

## Materials and Methods

### Participants

The sample included 75 male professional futsal players (age = 25.1 ± 5.1 years, body height = 182.3 ± 6.2 cm, body mass = 80.8 ± 10.4 kg) from seven futsal teams competing at the highest national level in Croatia, and Bosnia and Herzegovina (including the national champions in both countries for the preceding season). The participants were selected based on the following criteria: minimum 7 years of active involvement in futsal, older than 18 years of age, free from injury or illness, and regular performance of standard training for at least 3 weeks prior testing. The goalkeepers were not included in this investigation. For the purpose of this study, the total sample was divided into performance groups based on two criteria. The first criterion included clustering according to their status in the team, and thus players were identified as “starters” (i.e., first team; *n* = 35), and “non-starters” (i.e., substitutes; *n* = 40). This division was carried out by the head coach of each team. The second performance level clustering also included two performance levels, but players were observed as (i) top-level players (17 players) and (ii) high-level players (58 players). The top-level players were those who met at least one of the three following criteria: (1) they were members of senior-level national futsal team over the last 2 years; (2) they were members of the junior-level national futsal in the last competitive season (<18 years); and (3) they participated in the Union of European Football Association (UEFA) Futsal Champions League over the last 2 years, which is the highest competition level for futsal teams in Europe. The high-level players were those who were not grouped as the top-level players. All participants were tested over 3 weeks in September 2019 at the beginning of the competitive season. In this period, they trained 10–12 h (5–6 × ∼2 h) on the court to improve technical and tactical skills and 3 h (2 × ∼1.5 h) off-court in the gym to improve speed, strength and power. The participants were asked to refrain from any high intensity activity, tobacco, alcohol, and caffeine use and sleep deprivation for at least 2 days before the testing sessions. To stay properly hydrated, participants were allowed to drink water *ad libitum* in small amounts in each testing session.

The ethics board of the first author’s institution provided approval for the research experiment (Ethical Board Approval No: 2181-205-02-05-14-001). All participants were informed of the purpose, benefits and risks of the investigation. The participants voluntarily took part in the testing after they provided written consent. Sample size was estimated *a priori* using means and SDs from previous studies intended to evaluate the fitness status of professional level futsal players (Sekulic et al., 2019). Using G-Power software (version 3.1.9.2; Heinrich Heine University Dusseldorf, Dusseldorf, Germany), were estimated that 67 subjects would provide an appropriate sample size for paired-samples differences (*p* ≤ 0.05, power = 0.80).

### Procedures

Participants attended one familiarization session and two testing sessions. At the beginning of the familiarization session, the participants answered questions about their age, training, and health status and playing experience and level. Afterwards participants were familiarized to the physical capacity tests with special attention being paid to the futsal-specific agility tests (see details below). On the first testing day, anthropometrics were measured and CMJ, standing broad jump (SBJ), reactive strength index (RSI), sprinting over 5 and 10 m and the 20-yard generic CODS test (20 yards) were performed. On the second day, kicking speed with the dominant leg (Kicking-D) and non-dominant leg (Kicking-ND) was assessed, and the newly developed futsal-specific reactive agility RAG and futsal-specific CODS tests were performed. To minimize the variation in climatic and other conditions and to avoid diurnal variation all tests were performed in a sport hall on a parquet floor between 8:00 and 11:00. Prior to the assessment, participants performed standardized warm-up that included a 5-min self-paced running, followed by 5 min of dynamic stretching (e.g., high knees, lunges) and 5 min of futsal-specific high-intensity exercises (e.g., sprints and changes of direction speed with and without the ball). The rest between tests performed on the same day was standardized to 5–6 min.

### Variables

The anthropometric variables were measured with Seca stadiometers and scales (Seca, Birmingham, United Kingdom) and skinfold calipers (Holtain, London, United Kingdom) and included body height, body mass, and percentage of body fat (BF%). Body height (cm) was measured in bare feet to the nearest 0.1 cm. BMI was calculated by dividing body mass (m) by the squared body height (in meters). The BF% was calculated using body density (BD) according to the following formula: BD = 1.162–0.063 × log Σ4SF (where Σ4SF = sum of the biceps, triceps, subscapular, and suprailiac skinfolds). Body density was converted to body fat percentage: BF% = (4.95/BD−4.5) × 100 (Pehar et al., 2018).

The jumping abilities were estimated with the CMJ, SBJ, and RSI. The CMJ test was assessed with an Optojump system (Microgate, Bolzano, Italy), and the test is characterized by the stretch and shortening pattern of muscle function (Della Corte et al., 2020). In this test, the participant performs a maximum upward vertical jump after moving downward from an upright starting position with hands placed on the hips. In the SBJ, participants stand with their feet on the marked spot on the measuring scale (ELAN, Begunje, Slovenia). The length of the correct jump is recorded in cm from the line of reflection to the heel of the foot closest to the point of reflection (Sattler et al., 2012). The RSI is derived from the height jumped in a depth jump and the time spent on the ground developing the forces required for that jump [measured by Optojump device (Microgate, Bolzano, Italy)]. The starting position for the depth jump involved the subject standing upright on a 40-cm box. The subjects were instructed to step off from the box and to jump up maximally, attempting to minimize the contact time (Ebben and Petushek, 2010). Three jumping tests were performed, with a rest of 30 s between trials, and the best performance was used as the final achievement for each player.

Kicking-D and Kicking-ND were assessed with the shots taken from the 10-m spots that are used in futsal for accumulation penalty shots (Méndez et al., 2019). A Stalker-type hyper frequency radar instrument, with ±0.16 km/h margin of error (Stalker Professional Radar, Radar Sales, Plymouth, MA, United States) was placed 30 cm above the ground behind a goal. The reliability of this method for measuring kicking speed was previously confirmed (Sedano Campo et al., 2009). Participants needed to shoot standard-size futsal balls with both legs and were allowed to repeat attempts if the shot missed the radar. Five trials were performed, for each leg with a rest of 30–60 s, and the best performance was used as the final achievement for each player.

Sprint was measured with the Powertimer Newtest system (Oulu, Finland). The 5 m sprint and 10 m sprint were conducted together as the 5 m sprint result was a split time for the 10 m sprint. Three timing gates were used: the first was placed on the starting position, the second was placed on the 5-m mark, and the third was placed on the finish line (10 m). In the test, the participant is located in the high position to start, 1 m behind the start line, and starts the test arbitrarily. After the participant crosses the first gate, the time starts. The split time is noted after passing the second gate, and the time stops after the participant passes the third gate (Sekulic et al., 2013). Players performed the test over three trials with a rest of 2 min. The best (minimal) result was observed as the final achievement.

The 20-yard test was used as a measure of generic CODS. This procedure measures the ability of the participant to accelerate and quickly change direction at 180° (Sekulic et al., 2014). The test is organized with three cones placed on the same line with 5 yards between them. The timing gate is positioned at the middle cone, and the starting position of the participants is 0.5 m to the right in a lateral stance. The test starts when participants rotate their bodies to the left and trigger the time while passing the timing gate. The task is to run as quickly as possible to the cone on the left side, change direction and run to the opposite cone 10 yards away. After reaching that cone, the player changes directions again and runs toward the middle cone, stopping the time by passing the timing gate. Measurements were performed with a Powertimer Newtest system (Oulu, Finland), the test was conducted over three trials with 60 s of rest between trials, and the best performance was used in statistical analyses.

**FIGURE 1:**
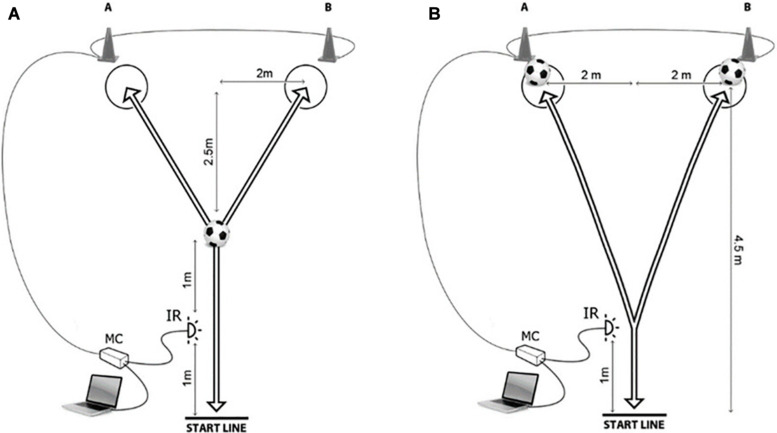
Testing of the futsal specific agility and change of direction speed with dribbling the ball **(A)** and without dribbling the ball **(B)**.

The futsal-specific CODS and RAG (FCODS and FRAG) were tested by the recently developed and presented futsal-specific CODS and RAG tests. The performance during FCODS and FRAG followed two procedures: (i) The participants had to touch the ball at the precise moment a change-of-direction occurred (FCODS_T and FRAG_T, respectively) and (ii) the participants dribbled a ball during the execution of each test (FCODS_D and FRAG_D, respectively). All tests had a Y-shaped pattern with the distances specified in Figure 1 (Figure 1A for tests that involved dribbling; Figure 1B for tests that involved ball touching). The timing for the FRAG tests began when the participants crossed the initial infrared signal. At that moment, a hardware module lit one 30 cm high cones (A or B). As no prior indication was provided for the FRAG tests, the participants had to quickly notice the specific light and react accordingly. Thus, the FRAG_D and FRAG_T performances were non-planned. For the FCODS tests, the participants had advanced knowledge on which cone would light up and therefore were able to preplan the movement template (Sekulic et al., 2019). Following the suggestions from previous studies, players were familiarized with the FCODS and FRAG tests over two practice sessions held 4–6 days before testing (Pojskic et al., 2018). In brief, all players were required to perform several trials and to demonstrate specific technique proficiency. The players were instructed to perform maximally concentrated tests and to identify the best individual movement strategies. Futsal-specific tests were later tested over five trials, with 1 min rest between trials, in a random order.

For the FRAG_D and FCODS_D, the participants were instructed to dribble a ball (Figure 1A) to a marked circle on the ground in front of the designated cone. The participants left the ball within the circle and changed direction to run back to the starting line as quickly as possible. For the FCODS_T and FRAG_T, the participants had to run to the ball, which was placed in front of the cone, touch it with the sole of the foot and run back through the infrared signal to stop the timer (Figure 1B). The FCODS_D and FCODS_T were performed over five trials consisting of three attempts. The FCODS was performed over four trials consisting of three attempts (A-B-A, B-A-B, A-B-A, and B-A-B), and players were informed of the upcoming scenario before each trial. The FRAG-D and FRAG_T were performed over five trials consisting of three attempts. Despite the fact that players performed FRAG_D and FRAG_T while not knowing the testing scenario in advance, all players were tested by same testing scenarios but in random order (A-A-B, B-A-B, A-B-A, B-B-A). The FCODS and FRAG tests used in this study were recently studied for reliability and validity, and the results were presented in detail elsewhere (Sekulic et al., 2019).

Measurement of the FRAG and FCODS tests was performed by a hardware device based on an ATMEL microcontroller (model AT89C51RE2; ATMEL Corp, San Jose, CA, United States). A photoelectric infrared sensor (E18-D80NK) served as an external time triggering input, and light emitting diodes were used as outputs. The photoelectric infrared sensor (Figure 1) has a response time of less than 2 ms and a digital output signal. The sensor distance for detection ranged from 3 to 80 cm with the ability to detect transparent objects. The sensor was connected with a microcontroller IO port (Figure 1). The device was connected to a PC operated on a Windows 7 operating system, as previously presented (Pehar et al., 2018; Pojskic et al., 2018).

### Statistics

Variables were checked for normality of the distributions by the Kolmogorov–Smirnov test, and descriptive statistics included means and standard deviations. The test-retest reliability of the variables was previously studied and reported in detail (please see previous text for references), and therefore, in this study, all tests were checked for intratesting reliability by calculation of the intraclass coefficient (ICC), and coefficients of variation (CV).

To define the differences between the groups, Student’s *t*-test for independent samples was applied and further analyzed using the magnitude-based Cohen’s effect size (ES) statistic with modified qualitative descriptors using the following criteria: <0.02 = trivial; 0.2–0.6 = small; >0.6–1.2 = moderate; >1.2–2.0 = large; and >2.0 very large differences (Hopkins, 2000).

Multivariate differences between performance-groups (starters vs. non-starters, high-level vs. top-level players) were analyzed by discriminant canonical analysis (DISCRA; forward stepwise model) and percentage of correctly classified cases was reported.

To identify the associations between variables, Pearson’s product moment correlation coefficients were calculated. The type I error rate of 5% (*p* < 0.05) was set *a priori* and was considered statistically significant. Stat Soft Statistica ver. 13.0 (Tulsa, OK, United States) was used for all analyses.

## Results

Table 1 presents descriptive statistics for all variable in total sample, and intra-testing reliability parameters for physiological tests. In general, all tests had appropriate intra-testing reliability with largest CV (i.e., lowest reliability) for RSI (11%), and FCODS and FRAG tests (8–10%).

**TABLE 1:** Descriptive statistics (mean, SD – standard deviation) and reliability parameters (ICC, intraclass coefficient; CV, coefficient of variation) for studied variables.

	**Mean**	**SD**	**CV**	**ICC**
Height (cm)	182.42	6.03		
Mass (kg)	80.88	11.57		
BMI (kg/m^2^)	24.25	2.75		
Body fat (%)	9.14	3.69		
CMJ (cm)	38.61	5.11	0.06	0.80
RSI (index)	145.97	37.45	0.11	0.75
SBJ (cm)	238.89	20.26	0.08	0.88
Kicking D (km/h)	105.39	6.09	0.09	0.79
Kicking ND (km/h)	92.85	9.30	0.10	0.80
Sprint 5 m (s)	0.98	0.09	0.07	0.91
Sprint 10 m (s)	1.71	0.11	0.05	0.93
20 yards (s)	4.65	0.26	0.06	0.90
FCODS_T (s)	2.11	0.19	0.09	0.79
FCODS_D (s)	2.50	0.26	0.08	0.78
FRAG_T (s)	2.42	0.24	0.09	0.77
FRAG_D (s)	2.63	0.25	0.10	0.77

*CMJ, countermovement jump; RSI, reactive strength index; SBJ, standing broad jump; Kicking D, kicking speed dominant leg; Kicking ND, kicking speed non-dominant leg; 20 yards, generic change of direction speed test over 20 yard distance; FCODS_T, futsal specific change of direction speed test without dribbling; FCODS_D, futsal specific change of direction speed test with dribbling the ball; FRAG_T, futsal specific reactive agility test without dribbling; FRAG_D, futsal specific reactive agility test with dribbling the ball.*

**FIGURE 2:**
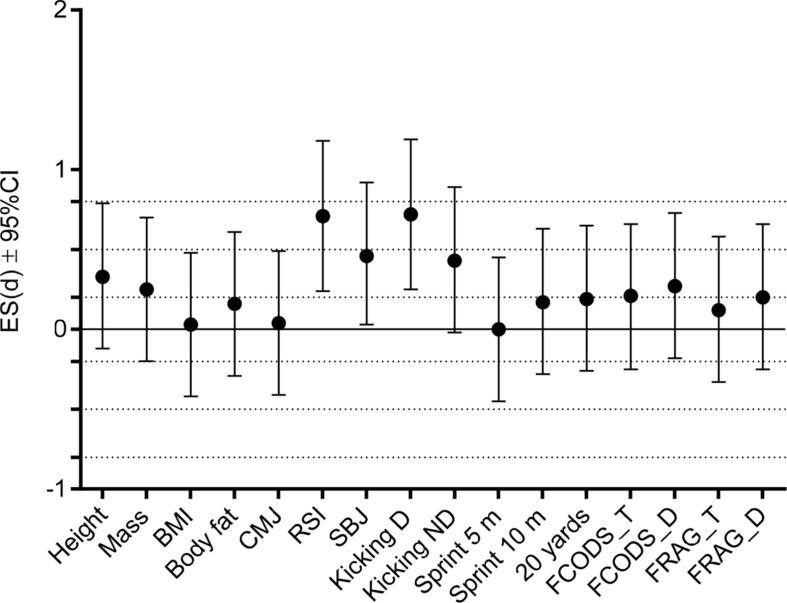
Effect size differences between starters and non-starters in studied anthropometric and physiological variables. CMJ, countermovement jump; RSI, reactive strength index; SBJ, standing broad jump; Kicking D, kicking speed dominant leg; Kicking ND, kicking speed non-dominant leg; 20 yards, generic change of direction speed test over 20 yard distance; FCODS_T, futsal specific change of direction speed test without dribbling; FCODS_D, futsal specific change of direction speed test with dribbling the ball; FRAG_T, futsal specific reactive agility test without dribbling; FRAG_D, futsal specific reactive agility test with dribbling the ball. Dashed lines present ES ranges (<0.02 = trivial; 0.2–0.6 = small; >0.6–1.2 = moderate; >1.2–2.0 = large differences).

Differences derived by *t*-test for independent sample between starters and non-starters are presented in Table 2. The ES differences between these performance levels are evidenced in Figure 2. In short, starters were significantly taller (small ES), achieved superior results than non-starters in RSI moderate ES), and in Kicking D (moderate ES).

**TABLE 2:** Univariate differences between starters and non-starters in studied variables (*t*-test for independent samples).

	**Starters (*n* = 35)**		**Non-starters (*n* = 40)**		***t*-Test**	
	**Mean**	**SD**	**Mean**	**SD**	***t*-Value**	***p***
Height (cm)	183.89	6.62	181.14	5.21	2.01	0.05
Mass (kg)	82.19	13.73	79.74	9.31	0.91	0.36
BMI (kg/m^2^)	24.21	3.00	24.29	2.56	–0.13	0.90
Body fat (%)	8.85	3.74	9.41	3.63	–0.66	0.51
CMJ (cm)	38.73	5.32	38.50	4.98	0.19	0.85
RSI (index)	159.25	37.86	134.35	33.39	3.03	0.001
SBJ (cm)	243.63	19.69	234.75	20.07	1.93	0.06
Kicking D (km/h)	107.01	6.46	103.98	5.44	2.20	0.03
Kicking ND (km/h)	94.66	9.96	91.28	8.50	1.59	0.12
Sprint 5 m (s)	0.98	0.10	0.98	0.08	–0.19	0.85
Sprint 10 m (s)	1.72	0.12	1.70	0.11	0.46	0.64
20 yards (s)	4.62	0.24	4.67	0.28	–0.96	0.34
FCODS_T (s)	2.09	0.17	2.13	0.21	–0.88	0.38
FCODS_D (s)	2.46	0.21	2.53	0.29	–1.06	0.29
FRAG_T (s)	2.40	0.23	2.43	0.25	–0.51	0.61
FRAG_D (s)	2.60	0.22	2.65	0.27	–0.73	0.47

*CMJ, countermovement jump; RSI, reactive strength index; SBJ, standing broad jump; Kicking D, kicking speed dominant leg; Kicking ND, kicking speed non-dominant leg; 20 yards, generic change of direction speed test over 20 yard distance; FCODS_T, futsal specific change of direction speed test without dribbling; FCODS_D, futsal specific change of direction speed test with dribbling the ball; FRAG_T, futsal specific reactive agility test without dribbling; FRAG_D, futsal specific reactive agility test with dribbling the ball.*

Multivariate differences calculated by DISCRA evidenced RSI and Kicking D as the strongest discriminators of the starters and non-starters, with 75% (*n* = 30) of non-starters, and 63% (*n* = 13) of starters being correctly classified (Table 3).

**TABLE 3:** Multivariate differences between starters and non-starters in studied variables (forward stepwise discriminant canonical analysis).

	**Canonical root**
Height	–0.41
CMJ	–0.04
RSI	–0.62
SBJ	–0.39
Kicking D	–0.45
Sprint 10 m	–0.09
Centroid: non-starters	0.53
Centroid: starters	–0.60
Wilks lambda	0.75
Canonical R	0.49
*p*	0.01

*CMJ, countermovement jump; RSI, reactive strength index; SBJ, standing broad jump; Kicking D, kicking speed dominant leg.*

**FIGURE 3:**
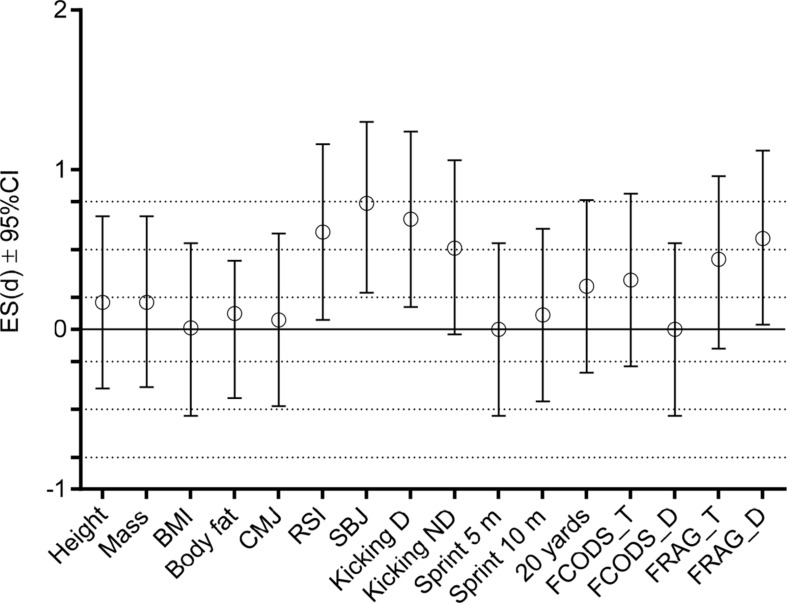
Effect size differences between top-level players and high-level players in studied anthropometric and physiological variables. CMJ, countermovement jump; RSI, reactive strength index; SBJ, standing broad jump; Kicking D, kicking speed dominant leg; Kicking ND, kicking speed non-dominant leg; 20 yards, generic change of direction speed test over 20 yard distance; FCODS_T, futsal specific change of direction speed test without dribbling; FCODS_D, futsal specific change of direction speed test with dribbling the ball; FRAG_T, futsal specific reactive agility test without dribbling; FRAG_D, futsal specific reactive agility test with dribbling the ball. Dashed lines present ES ranges (<0.02 = trivial; 0.2–0.6 = small; >0.6–1.2 = moderate; >1.2–2.0 = large differences).

Top-level players outperformer high-level players in RSI (moderate ES), SBJ (moderate ES), Kicking D (moderate ES), and FRAG_D (moderate ES) (Table 4 and Figure 3).

**TABLE 4:** Univariate differences between top-level players and high-level players in studied variables (*t*-test for independent samples).

	**Top-level (*n* = 17)**		**High-level (*n* = 58)**		***t*-Test**	
	**Mean**	**SD**	**Mean**	**SD**	***t*-value**	***p***
Height (cm)	183.84	7.88	182.01	5.38	1.11	0.27
Mass (kg)	82.44	9.88	80.43	12.06	0.63	0.53
BMI (kg/m^2^)	24.33	2.00	24.23	2.95	0.13	0.90
Body fat (%)	8.81	3.01	9.25	3.86	–0.43	0.67
CMJ (cm)	38.86	5.34	38.53	5.08	0.23	0.82
RSI (index)	163.42	38.04	140.86	36.02	2.24	0.03
SBJ (cm)	248.94	17.77	235.95	20.13	2.40	0.02
Kicking D (km/h)	108.82	6.21	104.38	5.72	2.76	0.01
Kicking ND (km/h)	96.41	9.59	91.81	9.03	1.82	0.07
Sprint 5 m (s)	0.98	0.09	0.98	0.09	0.11	0.91
Sprint 10 m (s)	1.71	0.12	1.71	0.11	–0.15	0.88
20 yards (s)	4.59	0.25	4.66	0.26	–1.10	0.27
FCODS_T (s)	2.07	0.15	2.13	0.20	–1.14	0.26
FCODS_D (s)	2.50	0.19	2.50	0.27	–0.01	0.99
FRAG_T (s)	2.35	0.19	2.44	0.25	–1.35	0.18
FRAG_D (s)	2.52	0.22	2.66	0.25	–2.07	0.04

*CMJ, countermovement jump; RSI, reactive strength index; SBJ, standing broad jump; Kicking D, kicking speed dominant leg; Kicking ND, kicking speed non-dominant leg; 20 yards, generic change of direction speed test over 20 yard distance; FCODS_T, futsal specific change of direction speed test without dribbling; FCODS_D, futsal specific change of direction speed test with dribbling the ball; FRAG_T, futsal specific reactive agility test without dribbling; FRAG_D, futsal specific reactive agility test with dribbling the ball.*

DISCRA successfully discriminated top-level from high-level players, with 94% of high-level players (*n* = 55), and 41% of top-level players correctly classified. The most significant discriminators were Kicking D, RSI, BJ, and FRAG_D (Table 5).

**TABLE 5:** Multivariate differences between top-level players and high-level players in studied variables (forward stepwise discriminant canonical analysis).

	**Canonical root**
CMJ	–0.04
RSI	–0.42
SBJ	–0.45
Kicking D	–0.51
Sprint 10 m	0.03
FCODS_D	0.00
FRAG_D	0.39
Centroid: top-level	0.53
Centroid: high-level	–0.60
Wilks lambda	0.71
Canonical R	0.53
*p*	0.001

*CMJ, countermovement jump; RSI, reactive strength index; SBJ, standing broad jump; Kicking D, kicking speed dominant leg; FCODS_D, futsal specific change of direction speed test with dribbling the ball; FRAG_D, futsal specific reactive agility test with dribbling the ball.*

Apart from logical and expected correlations between anthropometric indices, and various tests explaining the same capacity (i.e., correlations between sprinting variables, correlations between futsal specific CODS, and RAG performances), the correlates of tests previously reported to be highly discriminative between performance groups are particularly interesting. Specifically, RSI and FSRAG_D were negatively influenced by body fat (16 and 10% of the common variance, respectively). Also, RSI was positively correlated to Kicking D (10% of the common variance) (Table 6).

**TABLE 6:** Pearson’s correlation coefficients between studied variables.

	**Height**	**Mass**	**BMI**	**Body fat**	**CMJ**	**RSI**	**SBJ**	**Kicking**	**Kicking**	**Sprint**	**Sprint**	**20**	**FCODS**	**FCODS**	**FRAG**
								**D**	**ND**	**5 m**	**10 m**	**yards**	**_T**	**_D**	**_T**
Mass	0.58***														
BMI	0.13	0.88***													
Body fat	0.22	0.77***	0.81***												
CMJ	–0.14	−0.26*	−0.25*	−0.29*											
RSI	–0.12	–0.21	–0.18	−0.42***	0.36**										
SBJ	0.05	–0.20	−0.28*	−0.46***	0.63***	0.37**									
Kicking D	0.27*	0.12	–0.02	–0.16	0.25	0.35**	0.33**								
Kicking ND	0.28*	0.07	–0.09	–0.14	0.12	0.14	0.37**	0.61***							
Sprint 5 m	0.08	0.38***	0.42***	0.46***	−0.26*	−0.24*	−0.28*	−0.24*	–0.10						
Sprint 10 m	0.14	0.33**	0.32**	0.38**	−0.40***	−0.25*	−0.42***	−0.34**	–0.21	0.77***					
20 yards	0.05	0.46***	0.54***	0.63***	−0.39***	−0.46***	−0.46***	−0.30**	−0.40***	0.37**	0.40***				
FCODS_T	0.14	0.29**	0.25*	0.33**	–0.21	–0.31	−0.24*	0.05	0.09	0.06	0.09	0.43***			
FCODS_D	0.16	0.40***	0.38***	0.33**	–0.21	–0.21	−0.26*	–0.07	–0.08	0.16	0.13	0.51***	0.61***		
FRAG_T	0.06	0.27	0.26*	0.34**	–0.22	–0.22	−0.28*	–0.02	0.00	0.06	0.12	0.42***	0.66***	0.59***	
FRAG_D	–0.02	0.20	0.24*	0.35**	–0.12	–0.21	–0.22	–0.06	–0.07	0.09	0.11	0.40***	0.61***	0.63***	0.70***

CMJ, countermovement jump; RSI, reactive strength index; SBJ, standing broad jump; Kicking D, kicking speed dominant leg; Kicking ND, kicking speed non-dominant leg; 20 yards, generic change of direction speed test over 20 yard distance; FCODS_T, futsal specific change of direction speed test without dribbling; FCODS_D, futsal specific change of direction speed test with dribbling the ball; FRAG_T, futsal specific reactive agility test without dribbling; FRAG_D, futsal specific reactive agility test with dribbling the ball.

***p < 0.001, **p < 0.01, *p < 0.05.

## Discussion

The aim of this study was to determine the fitness status of professional futsal players and to define the factors that contribute to distinguishing performance levels. The main findings of the study are that (i) top-level players had a higher RSI, better results on the RAG test with a ball, and higher values of maximal kicking speed than high-level players and (ii) starters were taller and had a higher RSI and higher values of maximal kicking speed compared to the non-starters. Therefore, our initial study hypothesis can be partially accepted.

### Descriptions

The results of this study are in line with the results of a recent study revealing that futsal players from southeastern Europe are approximately 7 cm taller and 9 kg heavier than players from Italy, Spain, Brazil, and Australia who compete at a similar level of competition (Sekulic et al., 2020). Therefore, we may support conclusions and explanations of investigations in which similar results were evidenced for other sports. In brief, people from southeastern Europe – the former Yugoslav territory (including Croatia, Bosnia and Herzegovina) – are among the tallest European nations, and sport studies evidenced that these countries generally prefer taller athletes in the selection of team-sport athletes (Kondric̀ et al., 2012).

To efficiently perform specific high-intensity futsal movements (e.g., sprints, jumps, changes of direction), the ability of the lower body to exert force at high speeds (i.e., power output) is essential. In general, this quality is indirectly assessed by different jump and sprint performance tests with the CMJ and 5–20 m dash as the most frequently used tests (Naser and Ali, 2016). The results of this study indicated that players from this study had CMJ performance similar to that of futsal players from the first Brazilian league (38.7 and 39.2 cm, respectively) (Nakamura et al., 2016). Our futsal players performed similarly in 5 and 10 m sprint tests (0.98 and 1.7 s, respectively) to Brazilian futsal players (0.99 and 1.69 s, respectively) (Nakamura et al., 2016).

Shooting performance is one of the most important skills in futsal with speed and accuracy of the ball as the most important factors that affect shooting efficiency (Vieira et al., 2016). Shooting speeds of 108.8 km/h (top-level players) and 104.3 km/h (high-level players) reported in this work for Croatian and Bosnian-Herzegovinian futsal players were similar to those previously reported for Brazilian professionals, including members of the national team (99.7–109.1 km/h) (Milioni et al., 2016; Vieira et al., 2016). Given that the Brazilian national team is the third best in the world (according to the official FIFA rankings), we can say that players from the current study belong to the top tier of world futsal in terms of maximal kicking speed, sprinting, and CMJ performance.

### Differences Between Performance Levels

Top-level players had better reactive strength, as expressed by a higher RSI, than high-level players. Given that reactive strength is exhibited in movements consisting of a rapid eccentric contraction followed by a concentric muscle action, we can say that it is crucial in high-intensity activities that utilize the stretch-shortening cycle, such as sprinting, jumping, changing of direction speed, acceleration, and deceleration (Flanagan et al., 2008; Zatsiorsky et al., 2020). In support, our results indicated that players with higher RSIs (i.e., top-level players) performed better on the long jump test than players with lower RSIs (i.e., high-level players). Accordingly, RSI has been shown to have a strong relationship with CODS and acceleration ability in field sport players (Young et al., 2015). In other words, players with higher RSI are able to perform futsal-specific rapid motor actions more efficiently. Consequently, this ability may contribute to the success of the entire team by creating performance advantages over the opposing players during futsal matches. For instance, quicker players are more likely to outperform their opponents in situations in which they need to cover and defend certain space on the court or get to the ball first in trying to intercept the opponent’s pass (i.e., ball recovery) or receive it from a teammate in an open free space to create a scoring situation. Supportively, very recent study evidenced decelerations (one of the observed game workload variables) as a predictor of players’ physical profile (Ribeiro et al., 2020). Knowing that the ability to decelerate is directly related to eccentric muscular properties (similar to RSI), the importance of RSI in defining performance levels is additionally confirmed. It is well known that a higher level of reactive strength contributes to better agility performance (Alvarez et al., 2009). Therefore, it is not surprising that in addition to having better RSIs, top-level players outperformed high-level players in the FRAG_D. However, it is important to note that the FRAG_D applied in this study was quite complex and involved dribbling the ball. Thus, the FRAG_D results were not only affected by agility but also by futsal-specific skills. Such test characteristics emphasize the importance of futsal-specific motor proficiency in identifying performance levels in this sport and directly support the conclusions provided recently in a study in which the reliability and validity of the FRAG_D was explored in a sample of Croatian futsal players (Sekulic et al., 2019). Also, it has already been demonstrated that better players have greater skill in executing such actions than less skillful players (Farrow and Abernethy, 2003).

Top-level players achieved better results in maximal kicking speed than high-level players. The importance of this capacity was already discussed, and performance-level differences actually confirm the consideration of kicking speed as an important parameter of success in futsal (Ramos-Campo et al., 2016). Interestingly, although kicking speed can be affected by the power of the lower limbs and core, which corresponds to the maximal speed strength produced by the knee extensors, we did not find differences between top- and high-level players in CMJ (Bosco, 1999; Rodríguez-Lorenzo et al., 2016). Such a relative discrepancy (e.g., significant performance-level differences in kicking speed without significant differences in CMJ) can be explained by the weak correlation of vertical jump tests (i.e., CMJ) with maximal kicking speed (Rodríguez-Lorenzo et al., 2016), and several potential reasons exist for this result. Finally, the lack of association between CMJ and performance-levels in our study support even the recent considerations of Jiménez-Reyes et al. (2019) regarding the limited applicability of jumping tests in differentiation of the performance-levels in male futsal. First, CMJ is classified as slowing the stretch-shortening cycle (SSC) activity involving slow eccentric-concentric transition compared to the RSI, which is used to evaluate DJ performance as a fast SSC motor task (Ebben and Petushek, 2010). Therefore, it is logical to assume a weak correlation between CMJ as a low SSC activity and kicking performance as a rapid SSC activity. Second, although both activities require the SSC, the same muscles are not involved. In other words, CMJ involves the knee extensors and plantar flexors (e.g., quadriceps and gastrocnemius) as the prime agonist muscles, whereas kicking performance relies on both rapid pre stretching activity of the main hip flexors (e.g., the iliopsoas muscles) and the quadriceps muscles as the prime knee flexors and assisting hip flexors (Kellis and Katis, 2007). Finally, we might anticipate that the kicking technique (i.e., futsal-specific skill) that relies on the rapid SSC is a more important determinant of maximal kicking speed in professional players than the slow SSC activity *per se* evaluated by the CMJ performance. Because of the evident importance of kicking speed in futsal, this issue is additionally discussed later when we evaluate the correlates of kicking speed.

The results of our study did not indicate differences in anthropometrics and body composition indices between top-level and high-level players. In addition, no differences were found in generic CODS test (20 yards), and speed (sprint 5 m, sprint 10 m). The lack of differences between performance level groups in linear sprinting is not surprising. Interestingly, Sheppard et al. (2006) reported that a lower performance group of Australian football players outperformed the higher performance group in a 10 m straight sprint test but showed poorer performance in CODS and RAG tests (Sheppard et al., 2006). Similarly, recent studies suggested that performance level in futsal and football players cannot be differentiated by linear sprinting tests but only by futsal- and football-specific agility tests that include ball handling technique (Pojskic et al., 2018; Sekulic et al., 2019). This observation can be explained by the adopted sports-specific movement (i.e., running, acceleration, and deceleration) technique that includes lowering of the center of gravity, shorter strides, a sideways leaning posture toward the intended direction, and proper foot placement (Haugen et al., 2014). All of these factors together allow the lower-body muscles to apply sufficient and optimal lateral force to the ground, which in turn enables players to maintain balance and efficiently perform futsal-specific tasks that include many rapid changes of direction both with and without the ball (Haugen et al., 2014; Caetano et al., 2015; Santos et al., 2020). These observations together indicate that differences between the studied performance-levels are more closely related to (i) specific futsal skills and (ii) reactive strength, which enables players to efficiently execute futsal-specific movements, as already suggested (Sekulic et al., 2019).

In the present study, differences between starters and non-starters were fewer than between top-level and high-level players. These results may seem surprising because differentiation in these groups was regularly found to be highly discriminative in other team sports (Young et al., 2005; Gabbett et al., 2009). However, it should be emphasized that the decision on the starting line-up in futsal often depends on many different factors that can be highly complex (e.g., tactics, opposing team, game plan, importance of the match) and not directly related to conditioning capacities and futsal-specific skills. Therefore, relatively small differences between starters and non-starters should be contextualized by considering such specifics of futsal tactics. Regardless of all said, the better performance of starters in RSI and maximal kicking speed highlighted the previously discussed importance of these performances in futsal.

As such, the recent findings by Portuguese and Spanish authors (Santos et al., 2020) who investigated key performance indicators that discriminated all-star from non-all-star players during the Euro Cup 2018 Futsal (Slovenia) confirm the relevance of differentiating physical capacities (i.e., kicking speed, futsal-specific agility, and reactive strength) between starters and non-starters in this study. In brief, those authors found that all-star players had greater numbers for key pass accuracy and assists and achieved a higher number of goals with a better rate of shots on target during the matches. Moreover, the all-star group was shown to be better in defensive tasks (e.g., ball recoveries and challenges won). To outperform their opponents, futsal players must have not only excellent ball handling technique and anaerobic and aerobic endurance but also a high level of explosive power (e.g., kicking and running speed) and agility capacity. These capacities are especially crucial in defense to offense transitions when ball recovery requires both skillful players who understand the game and players who can predict the opponents’ action and can rapidly react by executing sprinting at the right moment and in the right direction, which in turn can enable ball recovery and create a scoring situation (Correa et al., 2014; Santos et al., 2020).

### Correlates of Performances

The results confirmed the importance of RSI, maximal kicking speed and futsal-specific agility performances in differentiation of performance levels in futsal. Although these features are highly complex (i.e., FRAG_D), partially genetically determined (i.e., RSI) and dependent on futsal-specific skills (i.e., kicking speed), the correlates of these physical capacities are important to discuss.

This study evidenced a negative correlation between body fat percentage and RSI and specific futsal agility tests. In other words, players with a higher body fat percentage had poorer reactive strength (i.e., lower RSI) and poorer futsal-specific agility. This conclusion can be elucidated by the “stop and go” nature of the test that permanently required players to change direction speed while overcoming their body inertia, which was more demanding if players had more fat as a non-functional ballast mass (Nikolaidis et al., 2015; Spiteri et al., 2015; Pojskic and Eslami, 2018). To be specific, following Newton’s second law, for a constant force, acceleration equals force per mass (e.g., a = F/m), which means that by reducing body mass (i.e., fat mass reduction), players could be able to accelerate their bodies at higher rate, and in turn, with higher body fat, mass acceleration could be slower. In other words, reducing the body fat mass of players could improve relative strength, which could inherently enable them to move more efficiently (e.g., accelerate and decelerate at higher rate) and perform futsal-specific tasks at superior level.

However, it is very important to emphasize that reducing body fat in futsal players could have multiple positive effects. First, excess body weight may be associated with an increased risk of fatigue and injuries (Martinez-Riaza et al., 2017). Therefore, a lower BF% can be observed as protective in injury prevention among futsal players. In addition, reducing body fat could likely have a positive effect on both reactive strength and futsal-specific agility. Specifically, such influence is suggested for other team sports in which jumping capacities and agility are important determinants of success (Sattler et al., 2015; Sisic et al., 2016; Pehar et al., 2018). Consequently, a decrease in body fat could improve the efficiency of execution of futsal-specific movements, ultimately increasing overall futsal performance.

Futsal players who were able to produce a higher maximal kicking speed were more agile. Although an explanation of the causality for this association is not within the scope of this paper, we attempt to indicate a possible background of the association and possible implications of this correlation in kicking performance. In short, throughout the kicking maneuver, the supporting leg (i.e., non-dominant leg) is responsible for the stability of the body during the swing phase and during the foot-ball contact phase (Rodríguez-Lorenzo et al., 2016). Generally, the main function of the supporting leg during shooting on the goal or passing is to absorb and resist the strong external forces to stabilize the body (Inoue et al., 2014). This type of work is characterized by negative force, and the muscles of the supporting leg (in running, this is called the loading phase) predominantly work eccentrically (Rorke, 1995). Consequently, the supporting leg should be well-trained in terms of eccentric strength, which directly influences kicking performance.

In addition, given that both kicking and sprinting performance are dependent on effective eccentric strength of the hip flexors and concentric strength of both the hip and knee extensors, a positive correlation was expected between them. In brief, to perform kicking at high speed (i.e., a quick shank and foot movement forward), it is important that the action is preceded by a rapid prestretch of the hip flexors and the knee extensors (i.e., fast backswing of the leg) (Kellis and Katis, 2007). In the same way, to quickly run, one must rapidly propel the legs forward by explosively lifting alternate legs (i.e., the thigh) off the ground and placing them forward. The optimal stride length, frequency and speed require rapid backswing of the leg that in turn results in prestretch of the hip flexors and the knee extensors. Therefore, both activities involve utilization of the SSC that enables augmented concentric (i.e., propulsive) muscular contraction of the legs. This action is possible because of ability of the musculotendinous system to store elastic energy when rapidly stretched (i.e., during eccentric muscular contraction) and to release it during propulsive movements (i.e., concentric muscular contraction) (Zatsiorsky et al., 2020). Additionally, rapid muscular lengthening is detected by the muscle spindles that induce the stretch reflex, which inherently increases the contraction strength of the agonist muscles during the concentric phase (e.g., take-off in running or jumping) (Zatsiorsky et al., 2020).

Similarly, it is well known that higher eccentric strength of the legs can contribute to better execution when performing change of directions (i.e., because it helps to efficiently decelerate the body, which allows faster performance of change of direction) (Brughelli et al., 2008; Kovacs et al., 2008; Jones et al., 2009; Spiteri et al., 2014; Chaabene et al., 2018). In addition, the supporting leg permits more reactive strength and better motor control of push-off actions, which allows faster 180° turns to be performed (Zouhal et al., 2018). Given that changes of direction at 180° are characteristic for the agility tests used in this work, it even explains the correlation between kicking speed and agility in our study. However, all previous discussions should be contextualized in light of a correlation between RSI and kicking speed and agility performance (i.e., RSI is significantly correlated with both performances). In brief, both kicking speed and agility performance are generally influenced by RSI (eccentric properties), and the association between agility and kicking speed should be at least partially observed as a result of statistical suppressor effect.

### Limitations and Strengths

The most important limitation of the study comes from its cross-sectional design. Therefore, although some causalities may be intuitively interpreted (body fat is almost certainly a cause of impaired performance and not vice versa), further studies are needed to evaluate the clear cause-and-effect among variables. Additionally, this study focused on motor performance, and indicators of aerobic and anaerobic endurance were not observed. Finally, although all teams were tested on the same floor (a standard wooden pitch where teams play official games), the measurement was not absolutely standardized.

This is one of the rare studies in which a relatively large sample of professional players were studied on a set of generic and futsal-specific physiological and anthropometric variables. All players were tested in a relatively short time span (e.g., in 3 weeks), which limited the possibility that seasonal variations significantly influenced the achievements. Finally, this is one study in which an international sample was observed and in which the pursuit of excellence was evaluated. Therefore, we believe that although it is not the final word on the topic, this study can contribute to knowledge in the field and promote initiation of further research.

## Conclusion

In conclusion, our findings suggest that reactive strength, kicking speed and futsal-specific RAG are important determinant of success (performance-level) in futsal. Knowing the level of the studied players (e.g., professional players of the highest national/international rank) we can conclude that these capacities can be considered essential qualities required for advanced performance in futsal. Collectively, findings of the study allow us to draw some conclusions which will hopefully improve even the training and conditioning in futsal.

Specifically, strength and conditioning coaches should focus on improving player ability to rapidly transit from eccentric to concentric muscular contractions emphasizing short ground contact time when performing different motor tasks (e.g., change of direction, sprinting, jumping). For this purpose, training programs that emphasize development of reactive strength of the leg extensors (e.g., plyometric training) would be particularly useful. Namely, application of such program will improve different conditioning capacities we have evidenced as being important determinants of success (i.e., reactive strength, kicking speed), and will consequently improve the qualities correlated to trained capacities (i.e., agility components).

Training programs in professional level futsal should include drills aimed on improvement of futsal-specific conditioning capacities, such as futsal specific RAG. Additionally, futsal strength and conditioning training should be targeted toward including futsal-specific skills (e.g., dribbling, shooting) in RAG drills. In development and application of such training, we may suggest progressive approach. Specifically, program should start with generic closed-skill drills and gradually progress to open-skill drills in which players are required to respond to various simple stimuli (e.g., a signal provided by a coach). Subsequently, exercises should include futsal-specific and decision-making “read and react” drills with real opponent(s) and match-like situations (e.g., “ball recovery drills,” “one-on-one play,” “small-sided games,” etc.).

Futsal training should focus on development of the eccentric strength of the hip flexors and knee extensors, which could improve the ability of the lower limbs to rapidly generate muscular force. This ability could provide players not only with the ability to accelerate and decelerate quickly in different movement directions but also to improve kicking speed, which is associated with a higher angular velocity of the knee joint and a faster approach of the player to the ball.

Special attention should be focused on proper learning of a CODS technique that includes lowering of the center of gravity, shorter strides, and proper foot placement. This improvement could enable players to move efficiently and to decelerate and stabilize the support leg, thus enabling greater muscle forces to be exerted when attempting to kick the ball.

Advanced lower-body strength relative to athlete body mass could be crucial in futsal play when players are continuously required to accelerate and decelerate while overcoming their body inertia. Supportively, our results indicated negative association between body fat percentage and several important futsal specific conditioning capacities. Therefore, professionals working with futsal players are encouraged to pay attention on body composition of their players. For such purpose (i.e., reduction of the body fat indices), specific nutritional regimes should be prioritized over extensive aerobic training (because of its potentially negative effects on power and sprinting capacities).

## Data Availability Statement

The raw data supporting the conclusions of this article will be made available by the authors, without undue reservation.

## Ethics Statement

The studies involving human participants were reviewed and approved by the University of Split, Faculty of Kinesiology (Ethical Board Approval No: 2181-205-02-05-14-001). Written informed consent to participate in this study was provided by the participants.

## Author Contributions

DS conceived and designed the study. IZ, MP, TM, and SV collected the data, performed the statistical analyses, and participated in drafting the manuscript. DS, TM, SV, and DN undertook the data analysis and interpretation. DS, HP, IZ, and MP gave an overview of the previous research and discussed the data. DS and HP did critical revision of the manuscript. All authors substantially participated in final manuscript versions, approved the submitted version, and agreed to be accountable for all aspects of the work.

## Conflict of Interest

The authors declare that the research was conducted in the absence of any commercial or financial relationships that could be construed as a potential conflict of interest.
